# Creation of a Systematic Interdental Brush Set Based on the Passage Hole Diameter (PHD) – An In-Vitro Study

**DOI:** 10.3290/j.ohpd.b5683229

**Published:** 2024-08-16

**Authors:** Caroline Sekundo, Friederike Ottensmeier, Stefan Rues, Hans Jörg Staehle, Manuel Pujades, Cornelia Frese

**Affiliations:** a Dentist, Researcher, Heidelberg University, Department of Conservative Dentistry, University Hospital Heidelberg, Im Neuenheimer Feld 400, 69120 Heidelberg, Germany; project administration, methodology, formal analysis, investigation, writing, reviewing and editing; b Dentist, Heidelberg University, Department of Conservative Dentistry, University Hospital Heidelberg, Im Neuenheimer Feld 400, 69120 Heidelberg, Germany; investigation, formal analysis, writing (original draft); c Engineer, Researcher, Heidelberg University, Department of Prosthodontics, University Hospital Heidelberg, Im Neuenheimer Feld 400, 69120 Heidelberg, Germany; methodology, investigation, writing, reviewing and editing; d Dentist, Professor, Heidelberg University, Department of Conservative Dentistry, University Hospital Heidelberg, Im Neuenheimer Feld 400, 69120 Heidelberg, Germany; conceptualisation, writing, reviewing and editing; e Researcher, Dentaid Research Center, Ronda de Can Fatjó, 10, 08290 Cerdanyola del Vallès, Spain; resources, methodology, writing, reviewing and editing; f Dentist, Professor, Heidelberg University, Department of Conservative Dentistry, University Hospital Heidelberg, Im Neuenheimer Feld 400, 69120 Heidelberg, Germany; supervision, project administration, writing, reviewing and editing

**Keywords:** interdental brush, systematic set, interdental hygiene

## Abstract

**Purpose:**

This study aimed to develop a systematic interdental brush set with size distribution based on the passage hole diameter (PHD), addressing existing gaps in size selection criteria for effective interdental cleaning.

**Materials and Methods:**

In the first step, an interdental brush set that ascends stepwise according to the PHD value was envisioned. The study was divided into three phases: (i) *in-vitro* determination of PHD values of a currently existing assortment on the market by 13 calibrated dental professionals, (ii) *in-vitro* assessment of forces during insertion, and (iii) creation and evaluation of new prototypes for missing or non-matching PHD sizes. Intra- and inter-rater reliability, assessed with the intraclass correlation coefficient (ICC), as well as insertion forces and PHD sizes at all stages were reported.

**Results:**

In the existing range, three interdental brushes fitting the desired PHD sizes were initially identified. Mean insertion forces between 0.3 and 1.7 N were documented based on raters’ PHD choices. Two additional rounds of measurements with prototypes adapted in diameter and shape were necessary, particularly for PHD values of 1.4, 2.3 and 2.6. High intra- and inter-rater reliability was observed throughout the study (ICC > 0.95), ensuring consistent evaluations. After three rounds of assessments, a prototype was successfully identified for each targeted PHD value in the systematised set, showcasing reliable sizing and insertion forces.

**Conclusion:**

Using a structured approach, a comprehensive interdental brush set was developed with reliable PHD sizing and moderate insertion forces. The verification of size reliability through measurements by dentists represents a novelty in development and underlines the importance of accurate brush size selection for optimal biofilm control. Whether a systematic set based on the PHD value offers added value for clinical practice, and at what intervals, must be demonstrated in further studies.

Owing to its anatomical location, the interdental space poses a greater challenge for cleaning than the exterior surfaces of teeth, necessitating specialised oral care interventions. Therefore, cleaning the interdental spaces is considered particularly important due to the insufficient coverage provided by routine toothbrushing practices.^7,16,17^ Biofilm control in this region becomes even more crucial for patients with loss of epithelial attachment and alveolar bone, often resulting from periodontal disease. To maintain stable periodontal conditions in the long term after systematic therapy, the often irregularly shaped interdental spaces and exposed root surfaces require individually adapted and regularly monitored biofilm control using optimally fitting, larger-sized interdental brushes.^14,27^ Another target group in this regard is the growing number of elderly individuals with either natural teeth, fixed or removable dentures, and/or implants.^23,24^ Even without previous periodontal disease, physiological ageing processes like bone resorption, enlarged interdental spaces, and exposed root surfaces are evident in this demographic. In the future, there will be a particular need for aids to clean larger interdental spaces. These aids must be chosen individually and precisely to meet patient needs.

Presently, numerous products such as dental floss, toothpicks, rubber or elastomer sticks and interdental brushes have flooded the market. While certain studies have identified a positive correlation with the use of dental floss,^5,18,32,33^ while others have argued insufficient proof of its benefits.^3,11^ Similarly, toothpicks have been found inadequate in ensuring optimal plaque reduction.^10^ Rubber sticks have been found to reduce gingival inflammation in young healthy adults,^9^ however, their use is not sufficient for larger interdental spaces, as mentioned above. Interdental brushes, in contrast, have achieved the best cleaning effect in several studies.^8,13,20–22,26,31,32^

Unlike dental floss or toothpicks, interdental brushes, due to their flexible wire core and filament components, are capable of conforming to the interdental space to achieve a comprehensive three-dimensional cleaning effect. An appropriately sized and shaped interdental brush has the capacity to reach areas such as subgingival spaces, periodontal pocket formations, and the concave regions of interproximal root surfaces. Besides minor gingival irritation, no severe adverse effects have been reported to date.^32^

Nonetheless, shortcomings exist in the proper utilisation and accurate size selection of interdental brushes, especially for larger and irregularly shaped interdental spaces. Despite the substantial significance of these tools in maintaining oral health, systematic methodologies are lacking, particularly concerning the choice of interdental brush size in daily practice.

Currently, the principal criterion for classifying the size of interdental brushes, according to the International Organization for Standardization (ISO) sizes 0 to 8, is the passage hole diameter (PHD) as outlined in the ISO standard for interdental brushes, ISO16409:2016.^12^ The ISO size is determined solely based on PHD values and not on other characteristics of the interdental brush. However, it must be noted that within ISO sizes 0 to 8, different PHD values are grouped together. This complicates the development of a systematically increasing brush set.

The PHD of an interdental brush is ascertained using a standardised measurement plate, whereby the brushes are pushed through holes of decreasing size using a ‘clinically relevant force’,^12^ which is not further defined. It varies depending on brush diameter, wire diameter, thickness, stiffness and layout of filaments. Despite this rather vague description, recent research^25^ has demonstrated a high level of intra- and inter-rater reliability amongst calibrated raters when determining the size of interdental brushes using the PHD, although larger sizes showed a higher degree of variance. However, only a quarter of manufacturers reported the PHD of their interdental brushes, whereas the ISO size was reported in a third of cases. The sizes offered on the market by a large number of manufacturers were also analysed, revealing significant inconsistencies in the size distributions of interdental brushes.25 These inconsistencies led to either an excess of similar sizes or significant gaps between available sizes. Such inconsistency is clinically relevant as the PHD size determines the smallest hole into which a brush can be inserted. Where an interdental space is slightly smaller than the available brush size, the brush may not fit or only fit with excessive force, risking tissue damage, or be too small to effectively clean the area. This issue, often overlooked in clinical practice due to a focus on brush diameter, was addressed in another study demonstrating the feasibility of creating a custom set with brushes from different manufacturers.^29^ However, the creation of such sets by regular dental professionals is impractical due to the labour-intensive process involved.

Therefore, this *in-vitro* study aims to bridge these gaps. Its objective is to develop a systematic set of interdental brushes, based on PHD, to provide a comprehensive range covering the most necessary sizes that facilitates selection for dental professionals. The adoption of interdental brushes with an accurate fit may potentially enhance cleaning efficiency, thereby offering advantageous therapeutic and/or prophylactic implications for oral health.

## MATERIALS AND METHODS

**Fig 1:**
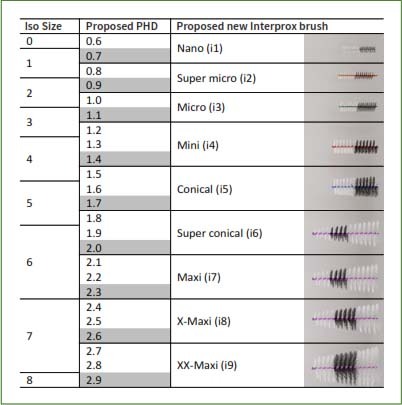
Proposed interdental brush set.

An interdental brush set was envisaged that included a brush specifically intended for every two PHD increments for the smaller sizes (PHD 0.6–1.1, approx. ISO 1–3), and one for every three PHD increments for the larger sizes (PHD 1.2–2.9, approx. ISO 4–8). For the smaller sizes, the objective was to use cylindrical brushes for precise insertion and cleaning efficacy, whereas conical, longer brushes were proposed for the larger sizes in order to accommodate the broader spectrum of PHD sizes (Fig 1).

The development of this systematic set was carried out in three phases: (i) Determination of the *in-vitro* PHD values by 13 calibrated dental professionals, beginning with the analysis of an existing assortment available on the market (Interprox Plus assortment, Dentaid, Barcelona); (ii) Assessment of the forces that occur *in vitro* during the insertion of the standardised measuring plate; and (iii) Development of new prototypes for those sizes that were previously inaccurately defined, or which needed to be smaller or larger for a systematic size distribution. This was followed by a re-evaluation of the newly created prototypes (repetition of phases (i)–(iii)). This process was intended to be repeated as many times as necessary.

### Phase I (Rater Measurements)

Dental staff working at the Department of Conservative Dentistry, University Hospital Heidelberg, were asked to participate in the study. To evaluate the size of the existing nine interdental brushes (Interprox Plus Nano; Interprox Plus Super Micro; Interprox Plus Micro, Interprox Plus Mini Conical; Interprox Plus Mini; Interprox Plus Conical; Interprox Plus Maxi; Interprox Plus X-Maxi Soft; Interprox Plus XX-Maxi) as well as the intra- and inter-rater reliability of the determined PHD, 13 raters were recruited. Ten dentists and three dental hygienists were calibrated in one-to-one sessions, whereby the contest of the ISO standard was conveyed^12^ and the stated application was demonstrated. Accordingly, the measuring plate had a thickness of 2.0 ± 0.1 mm and contained holes in 0.1 mm steps, through which eight samples of every brush were inserted in descending order. The test was terminated when reaching the smallest hole through which the sample passed completely without deformation with the above-mentioned ‘clinically relevant force’ determined by the rater. The PHD size of the nine brushes was evaluated in a randomised order, the interdental brushes were only referred to by colour, not by product name, to reduce bias regarding the correct size order. After 1 month, raters were asked to repeat their assessments.

### Phase II (Measurement of Insertion Forces)

**Fig 2:**
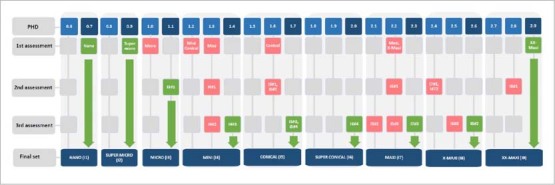
Developmental process of the systematic interdental brush set: sizes of the initial set and subsequent prototypes in relation to the proposal.

Laboratory analysis of the insertion forces was conducted using a universal testing device (Z005, Zwick/Roell) equipped with a 20N force transducer and testXpert III software. The forces exerted while passing the interdental brushes through the measurement plate were quantified. During the initial assessment of the preexisting interdental brush set, the median PHD was determined from the overall 26 individual rater measurements, which were then used to identify the correct PHD size for the brush insertion in the force determination process. This step was crucial to define the ‘clinically relevant force’ and to provide a basis for comparing subsequent prototypes. In the ensuing rounds conducted with the new prototypes, their respective forces during movement (insertion and extraction) were evaluated by threading the prototypes through the PHD size the prototype was assigned for, not for the size measured by the raters. A total of 10 samples of each interdental brush were tested as follows: First, zero force was determined before insertion of each interdental brush in the respective passage hole, then the interdental brush was lowered until 2 mm were inserted in the hole. Insertion with 6 mm/s speed ended with 2 mm of the interdental brush still located above the plate. Extraction to the starting position was also performed with a speed of 6 mm/s. In total, 21 insertion/extraction cycles were carried out and corresponding forces recorded. Given minor variations during the first cycle, only cycles 2–21 were assessed, during which the brush was inserted in alignment with the axis (Fig 2). Forces during insertion and extraction were evaluated separately in intervals, omitting the first 0.5 mm of displacement to exclude the switch in test force sign. Because there were insignificant differences between insertion and extraction forces, only insertion forces were reported.

### Phase III (New Prototype Development) and Repetition of Phases I–III

Interdental brushes that did not correspond to the requested PHD size were removed and new prototypes or modifications of existing interdental brushes for the missing PHD sizes were requested from the manufacturer.

After receiving the new prototypes, repeated measurements according to phase I and phase II took place. Both the examiners and the methods of measurement remained unchanged. In order to reduce measurement error, it was also investigated whether the measurement with and without brush handles caused any differences, as subsequent prototypes could not be fitted with handles due to the amount of time and cost necessary. The wires of the prototypes were therefore embedded in plastic for the measurements.

### Statistical Analysis

SPSS Version 24.0 was used to analyse the data. The intra- and inter-rater reliability was assessed using the intraclass correlation coefficient (ICC) for absolute agreement. Values above 0.75 were rated as good clinical reliability.6,19,30 Mean, SD and median PHD sizes, as well as mean and maximum insertion forces were reported at every stage.

## RESULTS

### Assessment of Existing Interdental Brush Sizes

Intra- and inter-rater reliability were examined to assess whether the first- and second-round measurements were consistent and whether there was general agreement among raters. Both dentists and dental hygienists achieved excellent intra- and inter-rater reliability (Table 1).

**Table 1:** Intra- and inter-rater reliability (n =13)

	ICC	95% confidence level		*P* value
		Lower bound	Upper bound	
Inter-rater reliability 1. Observation time (0M)	0.99	0.96	0.996	< 0.001
Inter-rater reliability 2. Observation time (1M)	0.99	0.97	0.997	< 0.001
Inter-rater reliability 1 + 2 Observation time	0.99	0.98	0.998	< 0.001
Intrarater reliability Mean ± SD	0.99 ± 0.008	0.87 ± 0.21	0.999 ± 0.001	< 0.001

**Fig 3:**
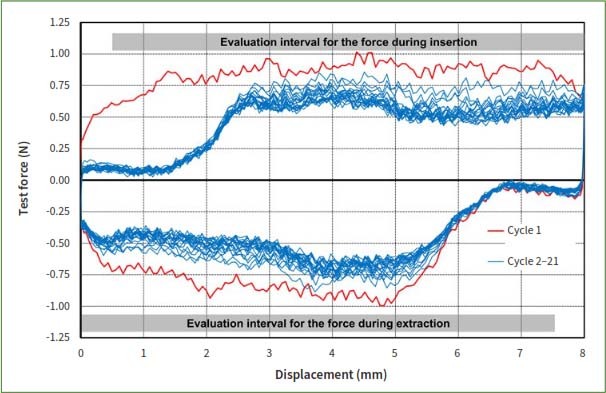
Exemplary force displacement diagram for an interdental brush with a length of 12 mm. The first test cycle differed in general from the other test cycles and was excluded from evaluation. To further exclude the switch in sign for the test force at the beginning of each insertion or extraction phase, the first 0.5 mm displacement was omitted from the respective evaluation interval.

The mean standard deviation and median of the raters’ measurements (phase I) at both timepoints (0 and 1 month) are shown in Table 2. Three interdental brushes (Interprox Plus Super Micro, PHD = 0.7; Interprox Plus Super Micro, PHD = 0.9 and Interprox Plus XX-Maxi, PHD = 2.9) could be directly assigned to the target set. These matched the desired PHD sizes (see initial set analysis, Fig 3).

**Table 2:** Mean PHD measurements by 13 dental professionals (both measurement timepoints)

1st round of assessment – initial interdental brush set analysis (Interprox Plus, Dentaid, Barcelona)											
	Nano	Super micro	Micro	Mini-conical	Mini	Conical	Maxi	x-maxi	xx-maxi		
Arithmetic mean	0.7	0.9	1.0	1.3	1.3	1.6	2.4	2.4	3.1		
Standard deviation	0.1	0.1	0.2	0.2	0.2	0.2	0.4	0.5	0.7		
Median	0.7	0.9	1.0	1.2	1.3	1.6	2.2	2.2	2.9		
2nd round of assessment – first prototypes											
	PT 0 / InterproxMaxi without handle	PT i3#1	PT i4#1	PT i5#1	PT i6#1	PT i6#2	PT i7#1	PT i7#2	PT i8#1		
Arithmetic mean	2.35	1.0	1.3	1.6	2.4	1.7	2.6	2.7	3.0		
Standard deviation	0.4	0.1	0.2	0.3	0.4	0.3	0.6	0.8	0.6		
Median	2.25	1.1	1.3	1.6	2.2	1.6	2.4	2.4	2.8		
3rd round of assessment – second prototypes											
	PT i4#2	PT i4#3	PT i5#2	PT i5#3	PT i5#4	PT i5#5	PT i6#3	PT i6#4	PT i7#3	PT i8#2	PT i8#3
Arithmetic mean	1.4	1.4	2.2	1.8	1.9	2.2	2.7	2.3	2.4	3.1	2.8
Standard deviation	0.27	0.22	0.51	0.3	0.3	0.5	0.7	0.5	0.5	0.9	0.6
Median	1.3	1.4	2.1	1.7	1.7	2.2	2.5	2.0	2.3	2.7	2.6

PT = Prototype.

During the *in-vitro* test (phase II), all investigated samples showed moderate mean insertion forces between 0.3–1.7 N when inserted into the median PHD deemed adequate by the 13 raters. The maximum insertion forces varied between 0.4 and 4.2 N (Table 3).

**Table 3:** Mean and maximum insertion forces of 10 samples each

1st round of assessment – initial interdental brush set analysis (Interprox Plus, Dentaid, Barcelona)				
Initial interdental brush (Interprox)	Fmax [N]		Fmean [N]	
	Mean value	SD	Mean value	SD
Nano	0.36	0.04	0.26	0.02
Super micro	0.61	0.10	0.32	0.04
Micro	0.82	0.16	0.54	0.04
Miniconical	1.95	0.30	0.87	0.08
Mini	1.02	0.16	0.63	0.06
Conical	1.42	0.25	0.87	0.09
Maxi	2.92	0.21	1.45	0.09
x-maxi	3.89	0.33	1.36	0.10
xx-maxi	4.21	0.46	1.71	0.09
2nd round of assessment – first prototypes				
Prototype i3#1	0.90	0.07	0.50	0.05
Prototype i4#1	0.74	0.03	0.52	0.01
Prototype i5#1	1.21	0.04	0.78	0.02
Prototype i6#1	4.61	0.80	2.24	0.21
Prototype i6#2	0.74	0.04	0.45	0.04
Prototype i7#1	4.48	0.38	1.84	0.19
Prototype i7#2	3.89	0.37	2.20	0.13
Prototype i8#1	3.84	0.42	2.23	0.17
3rd round of assessment – second prototypes				
Prototype i4#2	0.78	0.09	0.49	0.02
Prototype i4#3	0.86	0.05	0.51	0.01
Prototype i6#4	2.30	0.18	1.18	0.07
Prototype i7#3	1.97	0.07	1.05	0.04
Prototype i8#2	2.24	0.12	1.36	0.03
Prototype i5#2	1.11	0.09	0.61	0.03
Prototype i5#3	1.16	0.15	0.62	0.04

### Second Round of Assessment Prototypes

Mean, standard deviation and median of the raters’ measurements of the eight prototypes delivered (repetition phase I) at both timepoints (0 and 1 month) are shown in Table 2. Prototypes were adapted in size through changes made to one or more of the following features: Wire diameter, brush diameter, filament diameter. Two prototypes were tested for sizes i6 and i7. The interdental brush without a handle was measured similarly to its counterpart with a handle, with both showing comparable mean PHD sizes in the initial assessment of the set (mean PHD size = 2.25 vs 2.2, respectively; products: Interprox Plus Maxi without handle and Interprox Plus Maxi with handle). Handling of the prototypes for measurement without a handle was thus deemed adequate. The intra- and inter-rater agreement remained high (ICC > 0.98, 95%CI: lower bounds ≥ 0.96; upper bounds < 1.0; p < 0.001).

As described earlier, the new prototypes’ insertion forces were evaluated by threading the interdental brushes through the size the prototype was assigned for. This was done to better assess the new prototypes and compare their insertion forces with the ‘clinically relevant’ force measured when applying the PHD determined by the 13 raters in the initial assessment round.

In consequence, prototype i3#1 was rated as fitting (Fig 3), whereas prototypes i4#1, i5#1 and i6#2 were too small and prototypes i6#1, i7#1, i7#2 and i8#1 were too large. In particular, prototype #i7#1 had a high maximum insertion force (4.5 N), which is why further adaptation of prototype i7#2 was recommended. New prototypes were requested for sizes i4–7 after the 2nd round of assessment.

### Third Round of Assessment – Second Round of Prototypes

The third round of assessment was performed analogously. Mean, standard deviation and median of the raters’ measurements of the 11 new prototypes delivered (repetition phase I and II) at both timepoints (0 and 1 month) are shown in Table 2.

Only those prototypes that proved promising after the raters’ measurements were tested for their insertion force. The maximum and mean insertion forces ranged from 0.8 to 2.3 N and 0.5 to 1.4 N (Table 3). A prototype could be assigned to all searched and missing PHD values (Table 2, Fig 3). The intra- and inter-rater agreement was high (ICC > 0.95, 95%CI: lower bounds ≥0.93; upper bounds <1.0; p ≤ 0.02).

## DISCUSSION

Although the shared objective of the World Health Organization and many healthcare professionals is to enhance oral health by preventing caries and periodontitis through proper self-care, including interdental cleaning,^2^ there is a notable paucity of scientific research on the subject. Despite their role as a recommended routine tool for oral health maintenance,^8,13,20–22,26,31^ interdental brushes have received limited scholarly attention. Consequently, the development of interdental tools has been largely industry-driven, owing in part to the insufficient engagement of healthcare professionals in this area.

If a patient’s at-home oral hygiene routine appears effective and they maintain good oral health, there may be no immediate need to alter their interdental cleaning practices. However, should cariological or periodontal issues arise, professional advice on enhancing plaque control is necessary. This is particularly important in light of demographic changes. Due to successes in tooth preservation, a rising proportion of seniors and advanced elders retaining natural teeth is anticipated in the coming years. For this population, additional periodontal diseases, as well as fixed or removable dentures and implants, are often present, making consistent interdental hygiene critical. In these patients, enlarged interdental spaces are commonly observed, necessitating cleaning with individually selected and precisely fitting interdental brushes. In response to this need, a systematic set that fulfils these criteria is currently elusive. Therefore, in this *in-vitro* study, the multi-stage development of a new set of interdental brushes with stepwise increasing PHD sizes was carried out. The goal was to establish a uniform sizing system that covers the most commonly used size range for nearly all patients, thereby facilitating the selection of the right brush for professionals and private users.

Multiple challenges related to interdental cleaning using interdental brushes were addressed. Firstly, the PHD was employed as the metric for size definition. Although assessing PHD size is inherently challenging due to the absence of information regarding insertion forces in the ISO standard,^12^ it offers greater precision than ISO sizes. The latter can cover a wide range of PHD sizes, leading to considerable differences between neighbouring ISO sizes and posing a risk when brushes from different manufacturers are labelled with the same ISO size.^28^

The system developed in this study introduces a new level of standardisation and reliability by offering brushes in sizes based primarily on PHD, with ISO size considered secondarily. The ambiguity associated with determining the appropriate PHD size according to the ISO standard was mitigated by employing multiple professional raters, leveraging their clinical experience and utilising various measuring time points. The high levels of intra- and inter-rater agreement (> 0.9 ICC) corroborate the effectiveness of this PHD size determination method, that has also been applied in previous studies.25 However, it should be noted that disagreements among raters were more pronounced for larger brushes, suggesting that individual size selection for patients may exhibit greater variability in this range.

Since there are fewer different sizes of brushes available on the market for the larger segments, this can complicate the search for the appropriately sized interdental brush. While special brushes may be warranted for exceptional clinical cases, the range of PHD values offered in this set, from 0.7 to 2.9, aims to provide comprehensive coverage suitable for the majority of patients.

To date, there is no scientific consensus as to which forms are to preferred in interdental brushes. Both Rosing et al^20^ and Bock et al,^4^ in their studies involving 50 and 110 patients, respectively, determined that tapered and cylindrical interdental brushes offer similar levels of cleanliness. Larsen et al^15^ also found comparable overall cleaning effects, but noted that cylindrical brushes were more effective on lingual surfaces. Some studies have demonstrated that waisted interdental brushes are more effective at plaque removal *in vitro*.^1^ However, these brushes also exhibit higher insertion forces due to their thicker tips, making them challenging to apply.^25^ To counteract this effect, the anterior area of these interdental brushes has recently been equipped with thinner and more flexible side bristles.

Therefore, the practical application was considered in the proposed systematic set. For smaller interdental spaces, the challenge often boils down to a binary ‘can be inserted/cannot be inserted’ scenario, largely because trauma-free insertion is also influenced by wire diameter. Consequently, a close spacing of two PHD sizes was implemented up to a PHD size of 1.1. For larger sizes, greater variability exists. A brush can often fit within a range of sizes, yet the primary considerations then become whether the bristle length adequately covers the tooth’s surface and whether the insertion force is acceptable.

To address this variability, a spacing of three PHD sizes was implemented up to a PHD size of 2.9. Moreover, starting from a PHD size of 1.7, the brush heads were designed with a conical shape, with even longer heads for the largest sizes. This conical design not only facilitates insertion but also accommodates a wide variety of interdental space shapes. The insertion force can be modulated depending on how far the interdental brush is inserted. Moreover, longer side bristles were selected because, in addition to the PHD value, the length of the side bristles is an important parameter to assess the range of interdental brushes.

However, it should be noted that conical-shaped brushes are designed for application from both the vestibular and lingual/palatal sides. This may necessitate additional time and a higher level of skill, particularly for individuals who are inexperienced in using interdental brushes.

Further limitations include the study’s *in-vitro* design, which may not fully reflect clinical conditions. Nonetheless, the developed set is a first proposal based on theoretical and clinical considerations. It requires clinical verification in subsequent stages and modifications if necessary. Future research is essential to assess the application of this interdental brush set *in vivo* to better understand the relationship between oral anatomy, interdental brush usage, and cleaning efficacy as well as its acceptance by dental professionals and patients alike.

## CONCLUSION

This study encompassed multiple phases. Challenges in assessing the correct PHD sizes were addressed through the utilisation of a cohort of professional raters, while ensuring high levels of intra- and inter-rater agreement. Appropriate *in-vitro* insertion forces were defined based on these evaluations and were instrumental in assessing subsequent prototypes. Several prototypes underwent testing, with adjustments made to their sizes for optimal fit while also evaluating their insertion forces. Consequently, the resulting set exhibits reliable PHD sizing for the interdental brushes, complemented by moderate insertion forces.
